# Supplementary material for: Development of a computer-aided design software for the quantitative evaluation of aesthetic damage
Source: PLoS One. 2019 Dec 18;14(12):e0226322. doi: 10.1371/journal.pone.0226322 (PMC6919621; doi:10.1371/journal.pone.0226322)
Supplement: S1 Code site — 1. (ZIP) [file pone.0226322.s001.zip › [EESC_jr][Projeto]Relatorio_07_21/vendors/select2/docs/_includes/examples/data.html]

# Data sources

In addition to handling options from a standard `<select>`, Select2 can also retrieve the results from other data sources.

## Loading array data

Select2 provides a way to load the data from a local array.
You can provide initial selections with array data by providing the
option tag for the selected values, similar to how it would be done for
a standard select.

duplicate

{% highlight html linenos %}

duplicate
{% endhighlight %}

## Loading remote data

Select2 comes with AJAX support built in, using jQuery's AJAX methods.
In this example, we can search for repositories using GitHub's API.

select2/select2

When using Select2 with remote data, the HTML required for the
`select` is the same as any other Select2. If you need to
provide default selections, you just need to include an
`option` for each selection that contains the value and text
that should be displayed.

{% highlight html linenos %}
select2/select2
{% endhighlight %}

You can configure how Select2 searches for remote data using the
`ajax` option. More information on the individual options
that Select2 handles can be found in the
options documentation for `ajax`.

{% highlight js linenos %}
$(".js-data-example-ajax").select2({
ajax: {
url: "https://api.github.com/search/repositories",
dataType: 'json',
delay: 250,
data: function (params) {
return {
q: params.term, // search term
page: params.page
};
},
processResults: function (data, params) {
// parse the results into the format expected by Select2
// since we are using custom formatting functions we do not need to
// alter the remote JSON data, except to indicate that infinite
// scrolling can be used
params.page = params.page || 1;
return {
results: data.items,
pagination: {
more: (params.page \* 30) < data.total\_count
}
};
},
cache: true
},
escapeMarkup: function (markup) { return markup; }, // let our custom formatter work
minimumInputLength: 1,
templateResult: formatRepo, // omitted for brevity, see the source of this page
templateSelection: formatRepoSelection // omitted for brevity, see the source of this page
});
{% endhighlight %}

Select2 will pass any options in the `ajax` object to
jQuery's `$.ajax` function, or the `transport`
function you specify.
